# New Experimental Installation to Determine Adsorptive Properties of Magnesium Sulphate

**DOI:** 10.3390/ma13245652

**Published:** 2020-12-11

**Authors:** Oscar Banos, Sven Ohmann, Cornelia Breitkopf

**Affiliations:** Chair of Technical Thermodynamics, Institute of Power Engineering, Faculty of Mechanical Science and Engineering, Technische Universität Dresden, 01069 Dresden, Germany; Sven.Ohmann@tu-dresden.de (S.O.); Cornelia.Breitkopf@tu-dresden.de (C.B.)

**Keywords:** hydrates, adsorption, energy storage, refrigeration, heat pumps, composites, equilibrium, kinetics

## Abstract

Adsorption processes are of great interest in catalysis, material separation, and thermal management. In recent decades, adsorbents have been further investigated because of their applications in adsorption refrigeration, heat pumps, and thermal energy storage. Thus, there is an increasing need to determine the macroscopic properties of the adsorbent, specifically their adsorption/desorption capacity and speed, because these two factors determine the power and size of the corresponding adsorber. Many designs have been proposed, but there is still not a generally adopted technology for the analysis of those properties. In this paper, a novel instrument is described, which is capable of determining the macrokinetic properties of an adsorbent composite, with better control and higher accuracy than gravimetric, volumetric, or barometric installations, and lower price and complexity than spectroscopic installations. The design of the installation is detailed, highlighting the main challenges and critical factors. The two working modes of the installation are described, and one example is provided and analyzed for each of them.

## 1. Introduction

Because of their wide range of applications, adsorption phenomena are one of the most investigated processes. In the fields of adsorption refrigeration, heat pumps, and energy storage, the focus lies on the development of adsorptive composites or the design of adsorbers with high thermal conductivity and low inertia. This would increase their specific power and efficiency, reducing their size and operating costs [1]. Considering the increasing comfort standards regarding interior air quality, and the rising average global temperatures, adsorption technology is an immediate prospect of reduction of the dependence on fossil fuels [2]. The fact that adsorption systems can be powered with low quality heat makes them suitable to be combined with thermal solar systems or waste heat, reducing the demand of primary energy and improving the efficiency of industrial processes. Coated heat exchangers have been frequently proposed to achieve those goals, due to their simple construction and the reduced thermal resistance between the conductive surface and the adsorbent [3,4,5]. Expanded graphite is one of the most common materials in the production of adsorbent composites [6,7,8,9]. Metallic foams and metallic fibers have also been used as a substrate to increase the average thermal conductivity of the adsorber [10,11,12]. All these materials/designs have macrokinetic properties that differ from those of the pure adsorbent. In order to obtain representative data about their behavior under real conditions, it is necessary to use test benches that allow the analysis of samples whose size and geometry characterize the behavior of the adsorber.

The vast majority of instruments employed in the analysis of adsorption properties make use of one of the following principles: gravimetric, volumetric, barometric, or spectroscopic.

An example of a gravimetric installation can be found in the Fraunhofer Institute for Solar Energy Systems (ISE) (Freiburg, Baden Württemberg, Germany) [12]. It has a load cell inside a vacuum chamber which measures variations of the weight of the sample. They can measure an absolute water uptake of 55 g with a tolerance of ±2 g. The pressure in the chamber is controlled with the temperature of the evaporator/condenser. The fact that the load cell is placed inside the chamber limits the range of conditions and species that can be tested. Similar installations based on the same concept were used by Bonnacorsi et al. [7] and Poyet et al. [13]. The second reference reports a repeatability within ±2 µg with a sample of 2 g. This drawback can be overcome using a magnetic scale. The scale used in [6] has a sensibility of 0.1 µg for samples up to 200 mg. These scales allow a contactless coupling between the sensor and the sample through the wall of the reactor. The main disadvantage of this installation is its indirect heating of the sample, which reduces the heating rates that can be studied. Furthermore, the pressure in the installation influences the measurements, which requires corrections and increases the complexity of the installation. Another test bench based on gravimetric principles can be found in the work of Aidoun et al. [14]. It has a resolution of 1 g with a calibrated range of 1200 g. They present a design that measures the weight of the whole chamber rather than directly measures the weight of the sample. This concept avoids the need of using a magnetic coupling through the wall, reducing the complexity and cost of the installation. However, the connections of the pipes and sensors affect the measurements, reducing their reliability. Another gravimetric installation has been recently used by Palomba et al. for the characterization of adsorbent composites [15].

Barometric methods, such as the large pressure jump (LPJ) employed by Dawoud et al. [16,17], measure the pressure changes produced by the adsorption of vapor. In that case, the sample is maintained at a constant temperature in an evacuated vessel. When the pressure is suddenly increased by a supply of vapor from a secondary container, a pressure drop follows, caused by the adsorption of the refrigerant by the sample. The conditions of this experiment are different from those found in adsorption refrigeration, heat pumps, and energy storage, and its results are not easily extrapolated to those situations. For this reason, this principle is mainly employed in the characterization of adsorption phenomena in separation processes such as adsorption towers. A variation of this method is the large temperature jump (LTJ) presented by Aristov et al. [18]. In this case, the pressure is also measured, but adsorption is triggered by a jump in the temperature of the sample. Waszkievicz et al. [19] also presented a test bench based on pressure measurements, whose principle is more suitable for the conditions pursued in this work. The pressure and temperatures at different points of the adsorber were monitored. These were represented in a Clapeyron diagram to observe the cycle performed by the refrigerant. With the thermodynamic equilibrium properties of the pure adsorbent, it would be possible to determine the load state at each point, which would also allow for the transformation of the measurements into an evolution of the load against time. Another installation based on this principle can be found in Fraunhofer ISE and was used by Schnabel et al. [20] to test the performance of adsorbent composites. This installation was designed to test masses from 0.1 g to 2 g with a sensibility of around 0.03 g. To allow quick reactions, they installed an intermediate vessel between the evaporator and the reactor. This vessel contains a big volume of vapor, intended to attenuate the oscillations of pressure during tests. The main drawback of this installation is that it introduces a systematic error caused by the pressure drop due to the filling of the empty volume in the reactor.

The installation described by Jiang et al. [21] is based on volumetric principles. They determine the amount of adsorbed/desorbed vapor from the variation of the length of a liquid column. They employ a capacitive differential pressure transmitter that returns the pressure difference between the vapor just above the liquid column and at its bottom, which is directly related to the height of the column. To increase the sensibility of the installation, it is necessary to reduce the section in the evaporator/adsorber, which reduces the evaporation surface. With a transmitter with a sensibility of 0.02 mbar, this concept would need an evaporator with a diameter of 7 mm in order to have a sensibility of 1 µg of water. Another volumetric test rig is proposed by Huang et al. [22]. The level of liquid is measured with a magnetic displacement sensor. Similar layouts can be found in [23]. Iammak et al. [24] employed a combination of barometric and volumetric measurements, complemented with an energy balance, for the determination of the performance of the adsorber.

An example of spectroscopic installations is the test bench developed by Donkers et al. [25], which uses Nuclear Magnetic Resonance (NMR) to measure the water content inside the sample. The principle is based on the different relaxation times of water embedded in a crystal structure and water in a liquid solution. The Karlsruhe Institute of Technology (Karlsruhe, Germany) developed a sample technique called Inversed Micro-Raman Spectroscopy (IMRS) that substitutes the standard upright microscope in a Cofocal Raman Spectrometer by an Inverted Microscope, leaving the upper surface of the sample free for a better ambient control [26].

There are no literature data available that are comparable to our approach. The sensibility of the test rig described in this paper is higher than that achieved by gravimetric, barometric, and volumetric installations. Its cost and complexity are lower than those in spectroscopic test rigs. The development of innovative adsorbents will profit from the design presented in this paper, since it constitutes a more affordable concept for the characterization of materials and accelerate their commercialization.

## 2. Description of the Installation

The objective pursued with the design presented in this work was to obtain accurate and sensitive measurements of adsorption phenomena without the need of recurring to complex and expensive methods based on radiation spectroscopy. The equipment was conceptualized to allow the analysis of adsorbers under realistic working conditions, considering the framework of adsorbers intended for use in refrigeration, heat pumps, or thermal storage installations. Hence, the installation should allow the selection of the test pressure, and keep it constant during the experiment, while adsorption and desorption events are triggered by changes in the temperature of the sample. 

The installation was designed to be sensible to differences of adsorbates of less than 1 mg with water as the adsorbate. Its working principle is based on volumetric measurements. In order to allow the characterization of composites, the reactor was designed to hold samples with a representative size, capable of reproducing the conditions and geometries in real adsorbers. As the increase of the thermal conductivity is one of the key factors to obtain a powerful adsorber, the test bench should remove all those thermal resistances that are not intrinsic to the investigated material.

**Figure 1:**
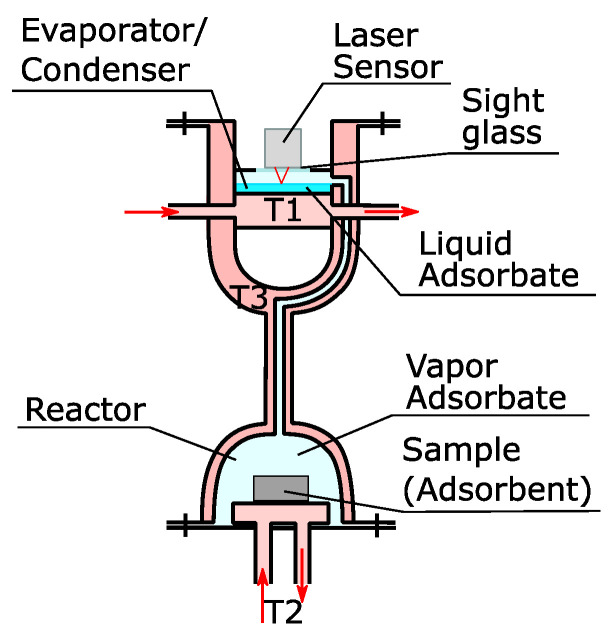
Scheme of the working principle of the new apparatus.

The basic structure of the installation can be seen in Figure 1. The pressure in the installation is controlled by the temperature in the evaporator/condenser, which is controlled by the fluid from a thermostat (T1). Fluid from another thermostat (T2) controls the temperature of the sample. The amount of vapor adsorbed or desorbed by the sample is determined by the displacement of the surface of the liquid in the evaporator/condenser. A laser sensor Keyence LK-H057 (Neu-Isenburg, Germany) with a range of 20 mm and a repeatability of 0.025 µm was placed on a sight glass to measure the displacements of the liquid surface. This measuring principle requires the equilibrium in the chamber to be maintained by evaporation or condensation on the liquid surface solely and not by condensation/evaporation on/from the walls. Most of the installations found in the literature rely on isolation or constant temperature chambers to avoid condensation. However, these are not optimal solutions. In order to avoid condensation on the walls, the whole installation was constructed with a double wall. The space between them is filled with the thermal fluid from a third thermostat (T3) that maintains the temperature 10 °C above the temperature of the evaporator/condenser. This has no effect on our control of the pressure because the vapor is in quasi-equilibrium with the liquid phase and variations of the pressure would be corrected by condensation/evaporation.

The chamber for the evaporator/condenser was designed in the way that could allow a fast response in case of small differences in the vapor pressure. If vapor is adsorbed/desorbed in/from the sample, it causes a variation of the vapor pressure from the equilibrium conditions. This deviation is compensated by the evaporation/condensation of liquid in the evaporator/condenser.

**Figure 2:**
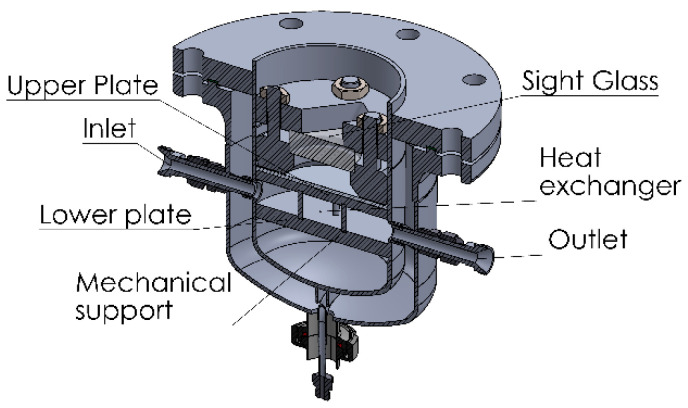
Double walled evaporator/condenser.

The enthalpy of evaporation/condensation results in a reduction/increment of the temperature of the liquid. As the pressure in the camber is controlled by the temperature of the liquid in equilibrium, it is necessary to ensure a sufficient heat transfer to the liquid film from the transfer fluid in the thermostat T1. The film of liquid adsorbate can be made as thin as required (max. 7 mm) to allow for a quick heat transfer. With a diameter of 150 mm, it can maintain the temperature of water within a range of ±2 K with adsorption/desorption rates of 1.6 mg/s. In order to reduce the time of reaction, the thickness of the upper plate was reduced, which required the addition of supporting bars (Figure 2). These bars transferred the pressure to the lower plate, whose thickness can be freely increased without affecting the thermal conduction to the liquid in the evaporator/condenser.

**Figure 3:**
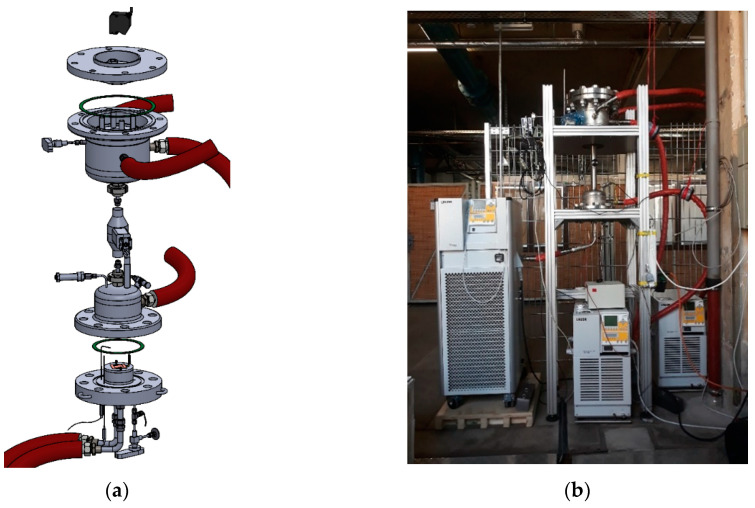
Explosion view (**a**) and photograph (**b**) of the macrokinetic installation.

The installation was designed in three main blocks (Figure 3). The modular design was chosen to facilitate maintenance and repairs. Modified compression fittings allowed a removable connection of pipes through the secondary wall. The reactor was designed to test pure adsorbents or composites with a maximum diameter of 100 mm and a maximum height of 50 mm. However, the design has enough flexibility to even test small prototypes of heat exchangers, with a maximum diameter of 130 mm and maximum height of 100 mm.

**Figure 4:**
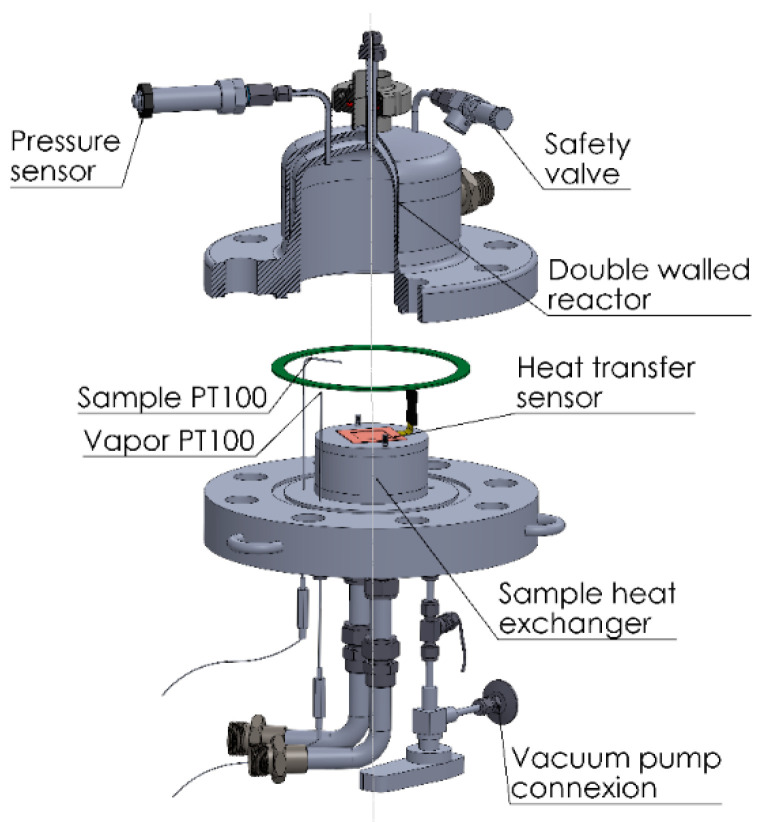
Reactor and sensors of the macrokinetic installation.

The reactor was equipped with three temperature sensors, one pressure sensor, and one heat transfer sensor (Figure 4). The connections for a safety valve, the heat exchanger of the sample, and a vacuum pump were also installed here. The heat transfer sensor (Captec enterprise, Lille, France) has a sensitivity of 5.18 µV/(W/m^2^), a sensing surface of 40 × 40 mm, and an integrated T thermocouple. The other two temperature sensors are two PT100 (Ahlborn, Holzkirchen, Germany) with a resolution of 0.01 K and a diameter of 0.5 mm for a fast response. They are intended for the measurement of the vapor temperature in the reactor and the temperature of the upper surface of the sample. Together with the measurement for the T thermocouple and the heat transfer, the gradient of temperature and the heat conductivity of the sample can be determined. Sufficient distance is maintained on the sides of the sample to allow for the installation of isolation that forces the heat and mass transport in one direction. This makes the experimental results representative of the behavior of the material, allowing the transfer of the data to theoretical models and facilitating the comparison with data from other adsorbers.

## 3. Results

**Figure 5:**
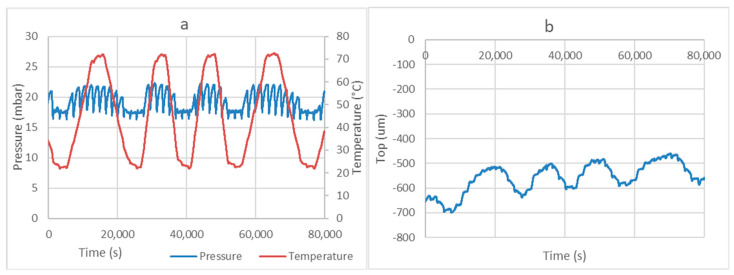
Example of a desorption–adsorption process with 20 g of MgSO4 with the evaporator at 15 °C (**a**). Diagram (**b**) represents the evolution of the position of the liquid surface in the evaporator, where “Top” is the position of the fluid surface with respect to the 0 point of the laser sensor.

The installation allows two working methods. The measurement of the adsorbed amount of vapor by the determination of the displacement of the liquid surface was already described in the previous section. Figure 5 presents the results of the measurements with a compact bed of MgSO_4_ powder. The diagram on the left presents the evolution of the temperature and the pressure. We see that the pressure remains constant with minor oscillations around the equilibrium pressure at the temperature of the evaporator (17.057 mbar for 15 °C). The oscillations of temperature result in adsorption/desorption processes, which are compensated by evaporation/condensation, respectively. This is indeed observed in the diagram on the right of Figure 5.

In addition, equilibrium tests to determine the thermodynamic properties of the adsorbent species can be conducted, following the principle described in [27]. The evaporator and reactor can be separated by means of a needle valve. This makes the reactor an isolated volume, where the corresponding adsorbent is in equilibrium with vapor adsorbate. A reduction in the temperature of the sample will cause the adsorption of vapor, which will reduce the pressure in the chamber until the equilibrium pressure is reached.

**Figure 6:**
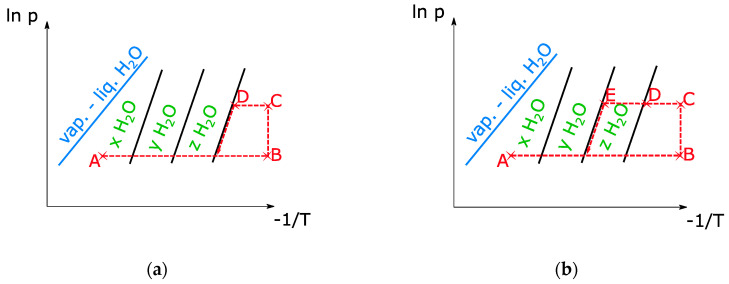
Clapeyron diagrams of equilibrium tests for H2O. Cycle for the determination of the lowest hydration step (**a**) and the second lowest hydration step (**b**).

**Figure 7:**
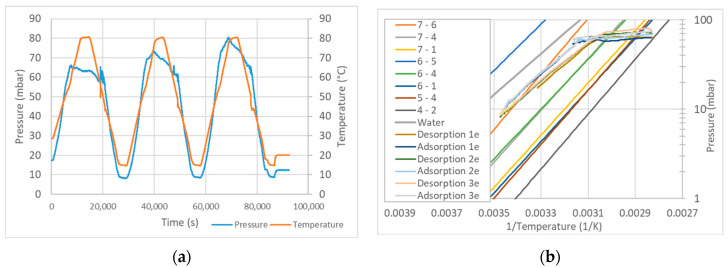
Example of a series of desorption-adsorption cycles with MgSO4 (**a**), compared with the van’t Hoff equation with data from Donkers et al. [28] (**b**).

In order to avoid uncertainties during the evacuation of the air in the installation, complete drying of the sample is performed prior to the adsorption-desorption cycles. The temperature is increased while the vacuum pump extracts the air from the chamber. At the same time, the pressure in the evaporator is set to the desired value. When state B (Figure 6) is reached, the evaporator and reactor are connected by opening the needle valve. Then the valve is closed again, and the temperature of the sample is decreased. When the equilibrium curve is reached (state D), the sample starts adsorbing vapor and the pressure decreases sharply. To measure other equilibrium curves, the valve is left open until the temperature decreases below the temperature in D. In the example in Figure 7, we see that the sample follows only one hydration step instead of the several steps predicted by the van’t Hoff equation. This is surely due to the poor thermal conductivity of the sample, which results in temperatures lower than those measured at the surface of the heat exchanger.

## 4. Overview and Conclusions

In order to reduce costs, the installation was designed employing as many normed parts as possible. The concept, however, is very versatile and can be adapted to other specifications. Smaller amounts of adsorbate can be surely detected with a narrower evaporator. The installation could also be adapted to higher adsorption rates, while maintaining the same tolerances for the deviation from equilibrium, at the cost of reducing its pressure rating.

The laser sensor is capable of measuring displacements of the liquid surface as small as 0.01 µm, which corresponds to a sensibility below 1 mg of water with the dimensions employed in this work. Increasing the number of customized parts, this sensibility could be reduced, mainly by reducing the diameter and the thickness of the sight glass. Optical sensors with higher sensibility could be used, and the displacement per unit of evaporated/condensed volume would be increased. We conclude that optical sensors allow higher sensitivity than other traditional alternatives (with the exception of spectroscopic installations)—e.g., if the sensor was to be substituted by a differential pressure transmitter, this should have a sensibility of around 10^−4^ Pa.

The design presented in this article constitutes a simple and cost-effective concept that will allow the characterization of adsorption materials with higher accuracy. Adsorber developers will profit from this design, facilitating the creation of new technologies and the expansion of the market of adsorption refrigeration systems, heat pumps, and energy storage systems.
